# New Insights Into the Role and Mechanism of Partial Epithelial-Mesenchymal Transition in Kidney Fibrosis

**DOI:** 10.3389/fphys.2020.569322

**Published:** 2020-09-15

**Authors:** Lili Sheng, Shougang Zhuang

**Affiliations:** ^1^Department of Nephrology, Shanghai East Hospital, Tongji University School of Medicine, Shanghai, China; ^2^Department of Medicine, Rhode Island Hospital and Alpert Medical School, Brown University, Providence, RI, United States

**Keywords:** renal fibrosis, myofibroblast, epithelial-mesenchymal transition, partial epithelial-mesenchymal transition, chronic kidney disease

## Abstract

Epithelial-mesenchymal transition (EMT) is described as the process in which injured renal tubular epithelial cells undergo a phenotype change, acquiring mesenchymal characteristics and morphing into fibroblasts. Initially, it was widely thought of as a critical mechanism of fibrogenesis underlying chronic kidney disease. However, evidence that renal tubular epithelial cells can cross the basement membrane and become fibroblasts in the renal interstitium is rare, leading to debate about the existence of EMT. Recent research has demonstrated that after injury, renal tubular epithelial cells acquire mesenchymal characteristics and the ability to produce a variety of profibrotic factors and cytokines, but remain attached to the basement membrane. On this basis, a new concept of “partial epithelial-mesenchymal transition (pEMT)” was proposed to explain the contribution of renal epithelial cells to renal fibrogenesis. In this review, we discuss the concept of pEMT and the most recent findings related to this process, including cell cycle arrest, metabolic alternation of epithelial cells, infiltration of immune cells, epigenetic regulation as well as the novel signaling pathways that mediate this disturbed epithelial-mesenchymal communication. A deeper understanding of the role and the mechanism of pEMT may help in developing novel therapies to prevent and halt fibrosis in kidney disease.

## Introduction

The progress of renal fibrosis – glomerulosclerosis and tubulointerstitial fibrosis – is characterized by an excessive deposition of extracellular matrix (ECM) components in the tubular interstitium. Fibrosis in the short term is an adaptive mechanism for repairing tissue damage, whereas persistent fibrosis after severe or repetitive injury eventually leads to renal failure as functional renal parenchyma is replaced by connective tissue (Farris and Colvin, 2012; Kaissling et al., 2013; Ovadya and Krizhanovsky, 2015; Djudjaj and Boor, 2018). Up to now, there is no specific therapy to halt progression of renal fibrosis. Moreover, the cellular and molecular mechanisms mediating fibrosis are still not well understood. A better understanding of the complex mechanisms and related cellular mediators of kidney fibrosis is urgently needed to develop new therapeutic approaches to prevent chronic kidney disease (Djudjaj and Boor, 2018).

During the pathogenesis of renal fibrosis, activated fibroblasts or myofibroblasts are generally thought of as the major type of matrix-producing cells (LeBleu et al., 2013). Similar to smooth muscle cells, myofibroblasts express α-SMA and secrete a pericellular matrix containing collagen and glycosaminoglycans. The origin of myofibroblasts in the kidney remains uncertain, but is considered to be derived from various sources: proliferation of resident fibroblasts (LeBleu et al., 2013), differentiation from local fibroblasts or pericytes (Lin et al., 2008; Humphreys et al., 2010), direct and complete transition from resident endothelial or epithelial cells through endothelial-mesenchymal transition (EndoMT) and epithelial-mesenchymal transition (EMT) (Carew et al., 2012). Some studies have suggested that bone marrow derived fibroblasts also contribute to the myofibroblast population (LeBleu et al., 2013). One of major controversies over renal interstitial fibrosis revolves around the relative contribution of activated tubular epithelial cells to the total myofibroblast pool.

EMT of tubular epithelial cells has been long considered a mechanism that promotes renal fibrosis. During that process, injured epithelial cells are activated and undergo a phenotypic conversion, acquiring the features of matrix-producing fibroblasts or myofibroblasts (Liu, 2010; Lee and Nelson, 2012). EMT was primarily identified in the kidney in a landmark article published in 1994 (Nadasdy et al., 1994) in which cells in renal interstitium with immunohistochemical features of tubular cells were found in end-stage kidney disease. In all tissues including kidney, key events during the process of EMT are summarized as follows (Kalluri and Weinberg, 2009; Fragiadaki and Mason, 2011; Prunotto et al., 2012; Lamouille et al., 2014; Loeffler and Wolf, 2015; Tennakoon et al., 2016): (1) dissolution of epithelial cell junctions and loss of cell polarity; (2) downregulation of epithelial proteins expression such as E-cadherin, zonula occludens-1 (ZO-1), and upregulation of mesenchymal marker expression including α-smooth muscle actin (α-SMA), vimentin, fibroblast-specific protein-1 (FSP1), and fibronectin (Gonzalez and Medici, 2014; Liu et al., 2018; Zhou et al., 2018); (3) reorganization of the cytoskeletal architecture and changes in cellular morphology with increased cell protrusions and motility; (4) acquisition of the ability to secrete factors to degrade extracellular matrix and exhibit invasive behavior (Liu, 2004; Lamouille et al., 2014; Loeffler and Wolf, 2015). Most studies applied co-immunostaining for markers of epithelial cells and, fibroblasts to detect the alternation of EMT transcriptional program. This does not, however, provide direct evidence of EMT (Iwano et al., 2002; Humphreys et al., 2010; Zeisberg and Neilson, 2010; LeBleu et al., 2013). Moreover, the exact percentage of fibroblasts that derive from tubular epithelial cells in a diseased kidney is entirely uncertain and remains under debate.

The controversy over the relative contribution of EMT to the total fibroblast or myofibroblast pool lasted many years. A study in 2013 used multiple genetically engineered mice to track the source of myofibroblasts. It was found that the source of myofibroblasts is split, with 50% arising from proliferation of local resident fibroblasts, 35% through differentiation from bone marrow, 10% from endothelial-to-mesenchymal transition. The contribution of EMT only occupies 5% of the total myofibroblast pool (LeBleu et al., 2013). Although a complete phenotype alternation from renal tubular epithelial cell to myofibroblast is rare, most studies provide evidence for a large population of tubular epithelial cells that co-express epithelial and mesenchymal markers. Recent studies found that the dedifferentiated TECs remain attached to the basement membrane after renal injury (LeBleu et al., 2013; Lovisa et al., 2016). This type of renal epithelial cells is considered to have undergone partial EMT (Grande et al., 2015; Lovisa et al., 2015). In this review article, we will discuss advances in our understanding of the role and mechanism of partial EMT in kidney fibrosis.

## Identification of Partial Epithelial-Mesenchymal Transition in the Kidney

In an early review examining the EMT hypothesis (Kalluri and Weinberg, 2009), it was suggested that epithelial cells undergo a “partial EMT” to acquire some of the phenotypic characteristics fibroblasts rather than converting to a “fully fibroblastic phenotype” (Kriz et al., 2011; Hajarnis et al., 2018). In 2015, two studies offered new perspective into the potential role of partial EMT in the development and progression of renal fibrosis (Grande et al., 2015; Lovisa et al., 2015). Both studies demonstrated that during the process of fibrosis, TECs expressed both markers of epithelial and mesenchymal cells while remaining attached to the basement membrane during fibrosis. Grande et al. (2015) demonstrated that *Snail1* induced partial EMT of renal tubular cells, which produces fibrogenic cytokines to promote myofibroblasts proliferation and macrophage recruitment. Lovisa et al. (2015) showed that the partial EMT program is associated with p21-mediated G2/M cell cycle arrest in proximal TECs. And inhibition of the EMT program by conditional deletion of *Twist1* or *Snai1* resulted in the maintenance of TEC integrity, decrease of immune cell infiltration and attenuation of interstitial fibrosis. These two observations demonstrated that without a complete conversion to fibroblast, a partial EMT is enough to induce TEC dysfunction by triggering cell cycle arrest, thereby promoting the secretion of profibrogenic cytokines and fibrosis.

## Phenotypic Alterations of Renal Tubular Cells With Partial EMT

During the process of partial EMT in kidney, epithelial cells experience series of changes, including cell-cycle arrest, metabolic alternation, and inflammation, that result in disturbed epithelial-mesenchymal crosstalk, leading to renal fibrosis.

### Cell-Cycle Arrest – Defective Repair and Regeneration

The cell cycle consists of four phases (G1, S, G2, M), each of which has specific characteristics. Cell division starts with the G1 phase. In the G2 phase, the cell prepares itself for mitosis by genetic material replication and protein synthesis. Genetic material and cellular components are distributed to two daughter cells during the M phase (Moonen et al., 2018). In G2/M arrest, cells are blocked from entering mitosis unless DNA replication or repair is complete, a cell size is large enough to divide and the cell has enough energy.

In acute kidney injury (AKI) models, such as ischemic reperfusion, acid toxic nephropathy, and obstructive mouse models, proximal TECs usually undergo G2/M phase cell-cycle arrest (Yang et al., 2010; Cianciolo et al., 2013; Canaud and Bonventre, 2015; Kumar, 2018). Chronic kidney disease is also associated with inappropriate cell cycle progression in TECs (Yang et al., 2010; Canaud and Bonventre, 2015). The number of tubular cells that undergo G2/M arrest is correlated with the degree of fibrosis (Yang et al., 2010; Kishi et al., 2019). Proximal tubular cells in G2/M arrest activate c-jun NH2-terminal kinase (JNK) signaling and upregulate profibrotic cytokine generation. The cells at G2/M phase have higher levels of COL4A1, ACTA2, TGF-β1, and CTGF expression (Yang et al., 2010; Canaud and Bonventre, 2015). Activation of signaling pathways such as TGF-β1 also triggers epithelial arrest at the G2/M phase of the cell cycle (Wu et al., 2013). Reversal of G2/M arrest rescues cells from the fibrogenic effects. This observation was further confirmed by several other groups (Cianciolo et al., 2013; Tang J. et al., 2013; Bonventre, 2014; Jenkins et al., 2014). In the setting of EMT, Lovisa et al. (2015) showed that after a fibrotic injury, TECs undergo a pEMT program and G2/M cell cycle arrest, which impairs cell regeneration and halts tissue repair, resulting in chronic fibrosis. Inhibition of pEMT in TECs protects kidney function by promoting cell cycle-dependent repair of the injured kidney. Therapeutic strategies targeting the G2/M check point are thought to be quite promising. Canaud and Bonventre (Canaud and Bonventre, 2015) suggested several approaches targeting cell cycle arrest. These include prevention of cells from activating downstream checks that trigger G2/M arrest, helping cells overcome G2/M checkpoints, facilitating apoptosis or selective depletion of senescent cells, and blocking pathways that are involved in production of profibrotic cytokines.

### Metabolic Alternation

The renal tubular epithelial cell is one of the most metabolically active cells in the body, and investigators are increasingly looking into the metabolic changes that occur during kidney fibrosis. Injured epithelial cells display dramatic metabolic rearrangements that affect their capacity to regenerate and influence fibrogenesis. Tubular cells have a high energy demand, producing ATP mostly by fatty acid oxidation (FAO) (Simon and Hertig, 2015). However, fibrotic kidneys exhibit lower expression of key regulators of FAO and higher intracellular lipid deposition in human and mouse models. Dysregulated FAO results in intracellular lipid accumulation and, inflammatory cell infiltration and cytokine secretion, and acceleration of interstitial fibrosis (Lovisa et al., 2016; Zhou and Liu, 2016). Lipid accumulation also promoted glucose-induced morphological changes of tubular epithelial cell, as well as the cytoskeletal switch including loss of E-cadherin and acquisition of α-SMA (Simon and Hertig, 2015). These results suggest that the decrease of FAO along with accumulation of lipids may promote development of EMT.

Currently, there is still no direct evidence indicating that the EMT results in metabolic alterations in the kidney. Nevertheless, Kang et al. (2015) demonstrated that TGF-β1 can induce remarkable depression of FAO through Smad3-mediated repression of the expression of PPARGC1a, one of the key FAO transcription factors in fibrotic kidneys. Given that TGFβ1-induced activation of Smad3 is also a key signaling event leading to EMT, this study also suggests that FAO depression may occur secondary or in parallel to EMT. Supporting this, it has been reported that upregulation of *Snail*, a major regulator of EMT, correlated with the downregulation of critical genes involved in FAO expression in hepatocellular carcinomas (Tanaka et al., 2013); pharmacological inhibition of FAO reverses Snail-mediated metabolic reprogramming *in vivo* and progression of breast cancer (Yang et al., 2020). Future studies are needed to elucidate the role of EMT in the regulation of FAO or other metabolic alteration in the kidney after fibrotic injuries.

### Pathological Secretome

Inflammation is widely described as a key promotor of kidney fibrosis. It is initiated as a protective response to acute injury. But when it persists, inflammation may contribute to fibrotic progression. Tubulointerstitial inflammation is observed in the early stage of different renal diseases. Apart from inflammatory cell infiltration, damaged TECs can be transformed into a secretory phenotype and act as proinflammatory mediators. The inflammatory response of TECs to injury is likely to be a key determinant in the development of interstitial fibrosis (Liu et al., 2018).

An early study (Abbate et al., 2002) explored the interactions between activated proximal tubular cells expressing the myofibroblast markers and peritubular cells, especially inflammatory cells. In remnant kidney models, α-SMA expression became evident in tubular epithelial cells at day 30, correlating with the ED1^+^ macrophages co-localization in peritubular areas in kidneys. This result suggests a relation between phenotype transition of epithelial cells and inflammatory cell recruitment. Grande et al. (2015) and Lovisa et al. (2015, 2016) examined the relationship between inflammation and EMT. They found that in fibrotic kidney, the immune response was attenuated after EMT inhibition with downregulated inflammatory cytokines. Lovisa et al. found that EMT inhibition resulted in a distinct reduction in immune cell infiltration in renal interstitium such as CD3^+^ T cells, CD8^+^ T cells, CD11c^+^ dendritic cells and macrophages. Grande et al. (2015) also observed a reduction in the presence of F4/80 macrophages, as well as lower levels of pNF-κB, cytokines and chemokines in tubular cells after EMT inhibition by deletion of *Snail*. In particular, the levels of M2-type macrophages, which are thought to be pro-fibrogenic during tissue repair, decreased evidently. Taken together, this suggests that blocking EMT could halt the related secretome of TECs and inhibit subsequent inflammation, serving as a potential approach to reduce fibrosis.

## Contribution of pEMT to Renal Fibrosis

Tubulointerstitial fibrosis is commonly seen during CKD progression (Fine et al., 1993; Eddy and Neilson, 2006; Zeisberg and Neilson, 2010). Studies showed that the phenotypic changes suggesting EMT correlate with the progression of kidney fibrosis. One study enrolled 83 kidney transplant recipients who had undergone allograft biopsies at 3 and 12 months after transplantation. Patients with vimentin and β-catenin expression in more than 10% of tubules at 3 months had much more severe interstitial fibrosis at 1 year. In addition, the presence of early phenotypic changes in TECs was associated with poorer graft function 18 month after transplantation (Hertig et al., 2008). These data suggest that epithelial phenotypic changes represent an “activated state” of the tubular cells that can lead to fibrosis.

The fibrotic interstitium is characterized by fibroblast and myofibroblast proliferation, inflammatory cell infiltration and tubular cell atrophy, as well as excessive accumulation of ECM (Prunotto et al., 2012). Historical data suggest that the progression of renal fibrosis might be the result of an altered epithelial-mesenchymal cellular crosstalk (Lewis et al., 1996; Johnson et al., 1998; Abbate et al., 2002; Yang et al., 2010). Well-balanced crosstalk between tubular epithelial cells and mesenchymal cells or inflammatory cells is essential to maintain a normal and healthy tubulointerstitium.

Experiments in the early 1990s showed the role of disturbed crosstalk between different cells during fibrogenesis. In the study of Johnson et al. (1998), cultured proximal tubule cells were found to be capable of stimulating fibroblast proliferation, collagen synthesis and MMP activity. This may occur through paracrine mechanisms that involve TEC-derived growth factors such as PDGF and TGF-β1. In cultured kidney epithelial cells, TGF-β1 stimulated profibrotic cytokines production and then promoted the proliferation and transition of pericytes to myofibroblasts (Wu et al., 2013). Bielesz et al.’s (2010) showed that activation of the Notch1 pathway in TECs contributes to tubulointerstitial fibrogenesis. TGF-β1 treated TECs induced Notch1 pathway activation, which not only promoted epithelial dysfunction, but also stimulated interstitial cell proliferation including myofibroblast proliferation. These results provide compelling evidence that TECs could regulate the behavior of neighboring myofibroblasts and fibroblasts in the interstitium. Disturbance of normal epithelial-mesenchymal unit crosstalk may be an initial cause of fibroblast and myofibroblast activation in the course of renal fibrosis.

Grande et al. (2015) showed that after partial EMT program activation, tubular epithelial cells were still integrated in the tubules and could express epithelial-related genes. The incomplete EMT program was still able to promote proliferation and activation of interstitial fibroblasts (Grande et al., 2015; Lovisa et al., 2015). This suggests that tubular cells undergoing partial EMT may produce some profibrotic cytokines/growth factors to induce renal fibroblast activation. In this regard, it has been reported that TGF-β1 treatment can induce cultured kidney epithelial cells to express several profibritic cytokines that stimulate the proliferation and transition of pericytes to myofibroblasts (Wu et al., 2013). Therefore, the fibrogenic response of EMT activation may not need complete transition of epithelial cells into myofibroblasts, and the cell with partial EMT acquire the ability to produce profibrotic cytokines/growth factors to induce renal fibroblast activation and renal fibrosis.

Increasing evidence indicates that the occurrence of EMT in renal tubular cells is associated with cellular senescence. Cellular senescence is mainly characterized by growth arrest and formation of a senescence-associated secretory phenotype that produces numerous profibrotic cytokines/growth factors, including TGF-β1 (Sosa Peña et al., 2018; Docherty et al., 2019). As mentioned, some growth factors and TGF-β1 not only induce renal fibroblast activation, but also stimulate the EMT response. Thus the cellular senescence-initiated EMT program may contribute to renal fibrosis. On the other hand, TGF-β1-induced partial EMT can also promote renal epithelial cells arrest at the G2/M phase of cell cycle and parenchymal damage in renal fibrosis. Therefore, it seems that EMT and senescence of renal tubular cells are intrinsically linked, leading to a vicious cycle in promoting chronic fibrosis. Currently, a causative link between these two processes and molecular mechanisms related to their reciprocal regulation in the fibrotic kidney are not yet been fully understood. It has been reported that induction of Twist or Snai1 expression in renal epithelial cells is sufficient to induce EMT and promote prolonged TGF-β1–induced cell cycle arrest in G2/M phase (Lovisa et al., 2015), suggesting that activation of these two transcriptional factors may play an essential role in mediating these two processes. Further studies are required to identify the signaling pathways that regulate senescence and EMT cross-talk as well as their potential co-targets.

## New Signals That Mediate pEMT and Subsequent Renal Fibroblast Activation

Defective crosstalk between epithelial cell and mesenchymal cell might involve multiple pathways of injury and repair. Epithelial cells communicate with interstitial fibroblasts by promoting activation and excitation of growth factors, such as TGF-β, CTGF, EGF, and FGF (Prunotto et al., 2012). In injured tubular epithelial cells, regular signaling pathways are disrupted and irregular pathways, such as Wnts and sonic hedgehog (SHH) (Kramann et al., 2015a,b), are activated. Activation of these irregular pathways could produce ligands of developmental pathways in pericytes or fibroblasts and trigger cell proliferation and fibrosis.

### Sonic Hedgehog (SHH) Signaling

Sonic hedgehog (SHH) signaling plays an important role in embryonic development, tissue regeneration and organogenesis. The disturbed activation of this pathway leads to pathological consequences, including the development of types of human tumors and fibrogenesis (Syn et al., 2009; Bai et al., 2016).

SHH expression is extremely low and difficult to detect in healthy kidney. It was activated in renal tubular epithelial cells in models of renal mass ablation, ischemia/reperfusion injury (IRI), and in renal tubules of biopsy specimens of CKD patients. When activated, it was predominantly localized in renal tubular epithelial cells. The sonic hedgehog pathway transcriptional effectors GLI1 and GLI2 are expressed in myofibroblast progenitors (Kramann et al., 2015a). Zhou et al. (2014) identified interstitial fibroblasts to be the targets of renal SHH signaling. Incubation with SHH or SHH overexpression promoted kidney fibroblast proliferation and stimulated expression of numerous proliferation-related genes. One study (Ding et al., 2012) also found that in ureteral obstruction model, SHH localized predominantly to the renal tubular epithelium. After administration of recombinant SHH protein, renal interstitial fibroblasts showed increased α-SMA, desmin, fibronectin, and collagen I expression, suggesting that SHH signaling participates in promoting myofibroblast activation and extracellular matrix accumulation.

SHH signaling might be involved in the process of EMT. Experiments demonstrated that injuries activated hedgehog signaling, resulting in tubular EMT and ECM production (Bai et al., 2014; Lu et al., 2016). Bai found that after ureteral obstruction, epithelial cells undergo G2/M cell cycle arrest and acquire a myofibroblastic phenotype. Meanwhile, the injury also stimulated activation of SHH signaling pathway. SHH-treated renal fibroblasts exhibited a fibrogenic response. Blockade of SHH inhibited fibroblast activation and improved ECM deposition (Bai et al., 2016). These results suggest that tubule-derived SHH activation might target interstitial fibroblasts and mediate epithelial-mesenchymal communication during EMT, thus promoting interstitial fibroblast proliferation and activation. SHH inhibition may be a potential therapeutic strategy to reduce fibroblast activation during kidney fibrosis (Syn et al., 2009).

### Wnt/β-Catenin Signaling

The Wnt family is a group of lipid-modified glycoproteins. The most studied Wnt signaling pathway in kidney is the Wnt/β-catenin signaling pathway, which plays an essential role in regulating nephrogenesis and pathogenesis of kidney diseases (Xiao et al., 2013).

Studies have shown a renoprotective effect of wnt/β-catenin in AKI models after either ischemic or nephrotoxic insults. Wnt/β-catenin protein was predominantly expressed in the kidney tubular epithelium. Tubule specific ablation of β-catenin aggravates injuries (Zhou et al., 2012). Nevertheless, chronic and persistent activation of this signaling pathway could lead to development and progression of CKD (Kawakami et al., 2013). Surendran et al. (2005) found that Wnt/β-catenin activation in UUO mice was associated with MMP-7, fibronectin, *Twist*, and c-Myc expression. Xiao et al.’s (2016) investigated the role of Wnt/β-catenin activation in IRI models. Moderate IRI led to acute kidney injury and transient Wnt/β-catenin activation, as a result, normal kidney morphology and function were restored. Severe IRI accompanied sustained and exaggerated Wnt/β-catenin activation resulted in the development of renal fibrotic lesions, suggesting that persistent Wnt/β-catenin signaling activation promoted fibroblast proliferation and renal fibrosis. Epithelium-derived Wnt/β-catenin signaling might disturb normal epithelial-mesenchymal crosstalk and thereby promote fibrosis (Xiao et al., 2016).

Recent evidence has demonstrated Wnt/β-catenin also contributes to the EMT process in tubular cells (Xiao et al., 2013). Hydrogen sulfide inhibits TGF-β1-induced EMT via Wnt/β-catenin pathway (Guo et al., 2016). CTGF induces tubular EMT through the activation of canonical Wnt signaling (Yang et al., 2015). Chronic cadmium exposure induces activation of the Wnt pathway and upregulation of EMT markers in mouse kidney (Chakraborty et al., 2010). It is indicated that Wnt/β-catenin signaling was activated during EMT process, and might alter the regular epithelial-mesenchymal cellular crosstalk.

## Epigenetic Regulation of pEMT in the Kidney

Epigenetic modification refers to stable heritable changes in gene expression without changes in DNA sequence. It is thought to contribute to the regulation of gene expression under different conditions (Rodriguez-Romo et al., 2015; Hewitson et al., 2017; Monteiro-Reis et al., 2019). The chromatin consists of histone proteins and the DNA wrapped around them. Epigenetic modifications involve selective unwinding of the double helix and exposure of the underlying genetic material (Tampe and Zeisberg, 2014). The modifications are well-balanced in normal cells. Small changes may lead to major consequences and result in cellular dysfunction or malignant outgrowth (Sun and Fang, 2016). Growing evidence suggests the epigenetic regulation of pro-inflammatory and pro-fibrotic gene expression, mediated by DNA methylation, histone modifications or microRNAs, is important in EMT and renal fibrogenesis (Wing et al., 2013; Rodriguez-Romo et al., 2015; Wanner and Bechtel-Walz, 2017; Lin and Wu, 2020).

### DNA Methylation

DNA methylation occurs at the 50-position of the cytosine ring (5mC) within CpG dinucleotides of gene promotors (De Chiara and Crean, 2016; Wanner and Bechtel-Walz, 2017). It is catalyzed by DNA methyltransferases (DNMTs). By inhibiting access of transcription factors to the binding sites or promoting the recruitment of methyl-binding domain proteins, gene expression is changed (Lee and Kong, 2016; Sun and Fang, 2016).

Carmona et al. (2014) found altered transcription of EMT-related genes in accordance with the dynamic changes of DNA methylation, reinforcing the role of the epigenetic mechanisms in the development of EMT. Direct methylation of certain transcription factors such as KLF4 and RASAL1 by Dnmt1 contributes to the progression of EMT in renal epithelial cells and is associated with fibrogenesis in the kidneys (Bechtel et al., 2010; Xiao et al., 2015). In arsenic-induced renal fibrosis, treatment with a DNA methylation inhibitor reversed the EMT properties of HK-2 cells, suggesting that the fibrotic changes are mediated by DNA methylation (Chang and Singh, 2019). There is still debate over the role of DNA methylation in renal fibrosis and EMT. By using comprehensive high-throughput arrays for relative methylation and TGF-β stimulation during EMT in AML12 cells, McDonald et al. (2011) showed no changes in DNA methylation.

### Histone Modification

Histone modification may be a key determinant of progression in renal diseases. The histone octamer is composed of a H3-H4 tetramer and two separate H2A-H2B dimers. The modifications, including acetylation, methylation, phosphorylation, ubiquitination, have been proposed to constitute specific biological outcomes (Sun et al., 2010; Wanner and Bechtel-Walz, 2017).

Recent studies show that histone acetylation through histone deacetylases (HDACs) activation mediated several physiological and pathophysiological changes in the kidney. Inhibition of histone deacetylase activity attenuated renal fibroblast activation and interstitial fibrosis in obstructive nephropathy (Pang et al., 2009). Histone 3 lysine 4 acetylation (H3K4Ac) is observed in the promoter regions of EMT marker genes, such as CDH1 and VIM. In hypoxia-induced EMT, HIF-1α induced histone deacetylase and then regulated EMT related gene expression (Wu et al., 2011; Wang et al., 2019). McDonald et al. (2011) investigated epigenetic modifications during EMT mediated by TGF-β, they found a reduction in the heterochromatin marker H3 Lys9 dimethylation (H3K9Me2), an increase in the euchromatin marker H3 Lys4 trimethylation (H3K4Me3) and in the transcriptional mark H3 Lys36 trimethylation (H3K36Me3). This suggests that during the process of EMT, specific chromatin domains across the genome were reprogrammed. In the UUO model, the number of individual histone markers, H3K9 acetylation (H3K9Ac) and tri-methylation (H3K9Me3) in proximal tubules and myofibroblasts, was increased 10 days after UUO (Irifuku et al., 2016; Hewitson et al., 2017). Targeting methyltransferases, such as disruptor of telomeric silencing-1 like (DOT1L) (Liu et al., 2019) and enhancer of zeste homolog 2 (EZH2) (Zhou et al., 2016), attenuates EMT and fibroblast activation by suppressing activation of multiple profibrotic signaling pathways. So, regulation of histone modification could be a strategy to interfere with the EMT process.

### MicroRNA

MicroRNA (miRNAs) are small, non-coding nucleotides that regulate diverse biological processes including aggressiveness of cancers and fibrosis. Altered microRNA biosynthesis or regulation contributes to pathological processes including kidney fibrosis (Tampe and Zeisberg, 2014; Wanner and Bechtel-Walz, 2017). Some suggests that microRNA crosstalk may influence epithelial-to-mesenchymal, endothelial-to-mesenchymal, and macrophage-to-mesenchymal transitions in the kidney (Srivastava et al., 2019).

Increasing evidence confirms various microRNAs are involved in the EMT process. Chen et al. (2015) found that after exposure to cyclosporine A (CsA), 46 miRNAs were significantly altered in proximal tubular epithelial cells, with alterations in EMT markers including vimentin and a-SMA. Several miRNAs are thought to be able to regulate different transcription factors including Snail, bHLH, and ZEB families are drivers of the epithelial plasticity leading to EMT (Díaz-López et al., 2015). The miR-200 family targets two important transcriptional repressors of the cell adherence (E-cadherin) and cell polarity genes, the ZEB1 and ZEB2 (Davalos et al., 2012; Tang O. et al., 2013). The hypermethylated or unmethylated status of miR-200 can be shifted corresponding to the EMT and MET (mesenchymal–epithelial transition) phenotypes of cells (Chen et al., 2015). Overexpression of miR-141 in HK-2 cells blocked the process of EMT by increasing E-cadherin and decreasing vimentin and FSP1 expression (Huang et al., 2015). And ZEB2 and cadherin-2 were directly regulated by miR-192 and -194 (Kim et al., 2018).

There are also some results suggesting a renal protective role of miRNA in renal injury. Zhao et al. (2017) found that miR-30c inhibited EMT through Snail1-TGF-β1 pathway, and attenuated diabetic nephropathy. In another study, researchers found that miR-152 overexpression inhibited TGF-β1-induced EMT in HK-2 cells (Ning et al., 2018). It is hard to identify which miRNA determines EMT initiation, and some have suggested unbalanced control of several miRNAs.

### Role of Extracellular Vesical Communication in the Kidney Injury, Recovery, and Fibrosis

Extracellular vesicles (EVs) such as exosomes are important mediators of cell-cell communication that act by transmitting proteins and genetic information. Vesicles incorporate molecules from their origin cells containing proteins, nucleic acids and lipids (Rigalli et al., 2020). A recent study demonstrated that exosomes are produced in renal proximal tubular epithelium following various fibrotic injury (UUO, ischemia/reperfusion or 5/6 nephrectomy) (Liu et al., 2020). Stimulation of cultured renal epithelial cells with TGF-β1 also increased exosome production, and the exosome purified from these cells are able to induce renal interstitial fibroblast activation (Liu et al., 2020). Conversely, pharmacologic inhibition of exosome secretion or depletion of exosome from the conditioned media blocked the ability of TGF-β1 stimulated renal epithelial cells to induce renal fibroblast activation; injection of tubular cell-derived exosomes also enhanced kidney injury and fibrosis (Liu et al., 2020). Given that TGF-β1 is a potent inducer of partial EMT and all three fibrotic injuries cited by Liu et al. (2020) can induce EMT, these results suggest that tubule-derived exosomes play an essential role in driving renal fibroblast activation and fibrogenesis. Since tubule-derived exosomes contain numerous miRNAs and growth factors that are beneficial to renal recovery and repair, TECs may also employ exosomes to regulate renal reparative responses (Borges et al., 2013; Guan et al., 2020). In addition, tubular-derived EVs may regulate inflammatory responses to affect renal injury, fibrosis and repair. In this regard, it has been reported that tail-vein injection of DM mice with EVs from HSA-induced HK-2 cells induces kidney macrophage M1 polarization and accelerate the progression of diabetic kidney disease through miR-199a-5p (Jia et al., 2019). Currently, there is still no direct evidence indicating whether renal epithelial cells undergoing EMT would promote EVs production and release. It has been reported that slug-mediated partial EMT in hepatocellular carcinoma cells can promote exosomal secretion of some proteins that can be considered as potential non-invasive biomarkers (Karaosmanoğlu et al., 2018). Based on the importance of tubular-derived EVs in regulating cellular communication and functional responses, it will be interesting to further elucidate the role and mechanism of extracellular vesicles in inducing partial EMT development and in mediating partial EMT regulated kidney injury and repair. Several recent review articles have described the production, regulation and therapeutic implication of extracellular vesicles in the kidney after acute and chronic injuries (Erdbrügger and Le, 2016; Morrison et al., 2016; Karpman et al., 2017; Lv et al., 2018).

## Conclusion

**FIGURE 1:**
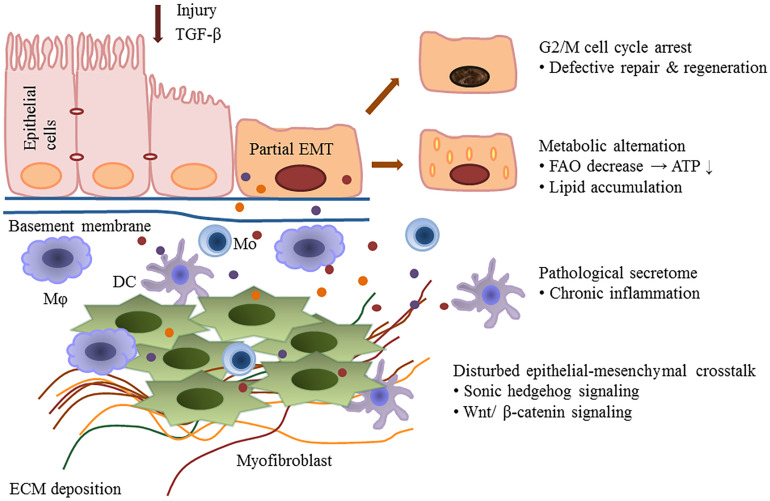
Partial epithelial-mesenchymal transition and renal fibrosis. After injury or TGF-b1 stimulation, epithelial cells undergo a partial EMT and remain attached to the basement membrane. This process leads to G2/M cell cycle arrest, impairing cell regeneration and tissue repair. Injured epithelial cells also display dramatic metabolic rearrangements, with great impacts on regeneration capacity and fibrogenesis. Epithelial cells secret cytokines and chemokines and release extracellular vesicles, followed by the recruitment of inflammatory cells including macrophages (M), monocytes (Mo), and dendritic cells (DC). The partial EMT also leads to a disturbed epithelial-mesenchymal crosstalk by overexpressing growth factors, activating several signaling pathways (such as the Wnts and sonic hedgehog signaling) and epigenetic reprogramming.

Kidney fibrosis is a multi-factorial and multi-cellular disease. All cell types present in the kidney including epithelial cells, endothelial cells, mesangial cells, as well as immune cells contribute to the progression of chronic kidney diseases. EMT has long been recognized as a main mechanism mediating renal interstitial fibrosis. In recent years, the proposal of partial EMT sets a novel insight on generation of epithelium dysfunction and tubulointerstitial fibrosis. According to recent studies, epithelial cell undergoes cell cycle arrest and metabolic alternation, inducing the infiltration of immune cells and disturbed epithelial-mesenchymal crosstalk, initiating and accelerating the process of fibrogenesis (Figure 1). Main findings related to partial EMT were listed in Table 1. More recent evidence demonstrates a role for epigenetic regulation of EMT. Considerable work still needs to be done to achieve more therapeutic possibilities.

**TABLE 1:** Main findings related to partial EMT.

**Main topics**	**Subtopics**	**Principle findings**	**References**
EMT	Concept of EMT	Injured TECs are activated and undergo a phenotypic conversion and acquire the features of matrix-producing fibroblasts or myofibroblasts. A complete phenotype alternation from TECs to myofibroblast is rare, most studies proved evidence for a large population of tubular epithelial cells that co-express epithelial and mesenchymal markers.	Liu, 2010; Lee and Nelson, 2012; LeBleu et al., 2013
Partial EMT	Concept of partial EMT	TECs expressed both markers of epithelial and mesenchymal cells while remained attached to the basement membrane during fibrosis. A partial EMT is sufficient to induce TEC dysfunction.	Grande et al., 2015; Lovisa et al., 2015; Hajarnis et al., 2018
Phenotypic alternation during partial EMT	Cell-cycle arrest	Partial EMT program is associated with p21-mediated G2/M cell cycle arrest, which impairs cell regeneration and halts the following repair, resulting in chronic fibrosis.	Canaud and Bonventre, 2015; Lovisa et al., 2015
	Metabolic alternation	The decrease of FAO along with accumulation of lipids was related to mesenchymal reprogramming of epithelial cells. Upregulation of *Snail*, a major regulator of EMT, correlated with the downregulation of critical genes involved in FAO expression.	Tanaka et al., 2013; Xu et al., 2014; Simon and Hertig, 2015
	Pathological secretome	EMT inhibition resulted in a distinct reduction in immune cells infiltration, as well as the lower levels of proinflammatory factors.	Grande et al., 2015; Lovisa et al., 2015, 2016
Epithelial- mesenchymal crosstalk	Disturbed epithelial- mesenchymal crosstalk	Fibrogenic response of EMT activation may not need complete transition of epithelial cells into myofibroblasts, and cells with partial EMT acquire the ability to produce profibrotic cytokines/growth factors to induce renal fibroblast activation and renal fibrosis. The cellular senescence-initiated EMT program may contribute to renal fibrosis.	Wu et al., 2013; Grande et al., 2015; Lovisa et al., 2015
	Signals mediating partial EMT	Sonic hedgehog signaling; Wnt/β-catenin signaling Tubule-derived SHH and Wnt/β-catenin signaling activation might target interstitial fibroblasts and mediate epithelial-mesenchymal communication during EMT.	Xiao et al., 2013; Bai et al., 2014, 2016; Guo et al., 2016; Lu et al., 2016
Epigenetic regulation of partial EMT	DNA methylation	Directly methylation of certain transcription factors contributes to the progression of EMT in renal epithelial cells and is associated with fibrogenesis in the kidney. DNA methylation inhibitor reversed the EMT properties.	Bechtel et al., 2010; Carmona et al., 2014; Xiao et al., 2015
	Histone modification	Histone modifications including acetylation, methylation activate EMT process and fibroblast activation by activation of multiple profibrotic signaling pathways.	Pang et al., 2009; Wu et al., 2011; Irifuku et al., 2016; Zhou et al., 2016; Hewitson et al., 2017; Liu et al., 2019; Wang et al., 2019
	microRNAs	Several miRNAs are able to regulate multiple transcription factors (i.e., Snail, bHLH, and ZEB), leading to EMT; Some other miRNAs contribute to renal injury by inhibition of EMT process.	Davalos et al., 2012; Tang O. et al., 2013; Chen et al., 2015; Díaz-López et al., 2015; Huang et al., 2015
Extracellular vesicles	EVs mediated cell-cell communication during EMT	Tubule-derived exosomes may play an essential role in driving renal fibroblast activation and fibrogenesis.	Borges et al., 2013; Guan et al., 2020

## Author Contributions

LS wrote the manuscript. SZ edited the manuscript. Both authors contributed to the article and approved the submitted version.

## Conflict of Interest

The authors declare that the research was conducted in the absence of any commercial or financial relationships that could be construed as a potential conflict of interest.
